# The Myth of the Stupid Believer: The Negative Religiousness–IQ Nexus is Not on General Intelligence (*g*) and is Likely a Product of the Relations Between IQ and Autism Spectrum Traits

**DOI:** 10.1007/s10943-019-00926-3

**Published:** 2019-10-05

**Authors:** Edward Dutton, Jan te Nijenhuis, Daniel Metzen, Dimitri van der Linden, Guy Madison

**Affiliations:** 1 Ulster Institute for Social Research, London, UK; 2 University of Amsterdam, Amsterdam, The Netherlands; 3 Parkville, Australia; 4 Institute of Psychology, Erasmus University Rotterdam, Rotterdam, The Netherlands; 5 Department of Psychology, Umeå University, Umeå, Sweden

**Keywords:** Intelligence, Jensen effect, Religion, IQ, Autism spectrum disorder

## Abstract

Numerous studies have found a negative relationship between religiousness and IQ. It is in the region of − 0.2, according to meta-analyses. The reasons for this relationship are, however, unknown. It has been suggested that higher intelligence leads to greater attraction to science, or that it helps to override evolved cognitive dispositions such as for religiousness. Either way, such explanations assume that the religion–IQ nexus is on general intelligence (*g*), rather than some subset of specialized cognitive abilities. In other words, they assume it is a Jensen effect. Two large datasets comparing groups with different levels of religiousness show that their IQ differences are not on *g* and must, therefore, be attributed to specialized abilities. An analysis of the specialized abilities on which the religious and non-religious groups differ reveals no clear pattern. We cautiously suggest that this may be explicable in terms of autism spectrum disorder traits among people with high IQ scores, because such traits are negatively associated with religiousness.

## Introduction

Since the 1920s (e.g. Gilkey 1924; Howells 1928), a substantial number of studies have found a weak but consistent negative relationship between religiousness and IQ. Meta-analyses have shown that this relationship is in the region of − 0.2 in the general population, and around − 0.1 among college students (e.g. Zuckerman et al. 2013; Dutton 2014). Similar negative correlations are also found between religiousness and diverse proxies for IQ, such as educational level and income (Meisenberg et al. 2012). The association is greater at the level of countries, with estimates of national average IQ being correlated with national average levels of religiosity at about − 0.6 (Lynn and Vanhanen 2012). The negative religiousness–IQ nexus is found within most countries, with only a small number of exceptions (Meisenberg et al. 2012). The association can be found both among young and elderly samples (Ritchie et al. 2014). The more pronounced in its religiosity a group is, the lower the average IQ its members tend to possess (Lewis et al. 2011; Nyborg 2009), whereas members of high IQ organizations, such as the Royal Society, tend to be overwhelmingly atheist (Larsen and Witham 1998). The evidence for a negative association between religiousness and IQ is thus robust.

The precise causes of this relationship are less clear. Nyborg (2009) argues that people are attracted to different ways of understanding the world based on their ability to deal with complexity. Science is too complex for those with lower intelligence, who therefore resort to the simpler explanations and life-guiding rules that religions typically provide. Dutton (2014) suggests that the elevated reasoning ability entailed by higher intelligence fosters the ability to see through what he regards as the fallacious arguments for the existence of God, and to therefore conclude that there is no God in the absence of supporting evidence. Kanazawa’s (2012) Savanna–IQ Interaction hypothesis rests on the assumption that our ancestors were most strongly adapted to the ecology of the Savannah. Accordingly, this is our ‘evolutionarily familiar’ environment, because, having spent so long in it, this ecology selected certain evolved modules useful for dealing with the recurrent problems found in that ecology. One set of modules that this ancestral environment selected for might have been those associated with the generation of social behaviors undergirding religiousness. As humans left the Savannah, they began to encounter evolutionarily novel problems that these modules could not solve, and developed higher intelligence that could successfully solve these novel problems. Thus, intelligence became associated with ‘evolutionarily novel preferences’, such as atheism. There are several conceptual and empirical problems with this hypothesis, highlighted by Dutton and van der Linden (2017). One is that evolution continued, and maybe even accelerated, during the Holocene (Cochran and Harpending 2009; Woodley of Menie et al. 2017) which the Savanna–IQ Interaction hypothesis assumes is not the case. Dutton and van der Linden (2017) proposed the *Intelligence Mismatch Association Model*. It suggests that one aspect of intelligence is the ability to rise above our evolved cognitive biases, as this allows us to better solve new cognitive problems by being more open to unusual potential solutions. The difference between the original Savanna–IQ Interaction hypothesis and the Intelligence Mismatch Association model is that the latter applies to any evolved behavioural or cognitive repertoire, thereby avoiding the alleged subjective distinction between ‘evolutionarily novel’ and ‘evolutionarily familiar’ problem content. Dutton and van der Linden’s argument is simply that humans have certain cognitive biases, intelligence is associated with rising above them in order to solve problems more flexibly, and as religious belief is a cognitive bias, intelligence should be negatively associated with it.

However, all these models assume that the negative religion–IQ nexus is predominantly related to *general intelligence* (*g*), as they all assume that religious people are less intelligent than atheists. The *g* factor is likely the construct that best represents the heritable components of intelligence, and hence that which evolution has most strongly acted upon historically in human, and more broadly, primate populations, as indicated by comparative phylogenetic analyses of the evolutionary rates for different cognitive abilities, where there are strong indications that such rates increase in proportion to the loading of these abilities onto a Big-G factor of inter-specific cognitive ability (Fernandes et al. 2014). An IQ test score is typically the sum of several subtests that measure specific cognitive abilities and will therefore include these specific abilities. A proper measure of *g* should rather ignore the specific abilities, but capture the variance that is common among them. This is achieved by factor analysis, the first unrotated factor of which typically explains 50–60% of the variance among all subtests across a number of individuals. Factor analysis also yields the factor loadings for each subtest, corresponding to the proportion of the common variance between the subtest and *g*, referred to as its *g* loading (Jensen 1998). The Jensen effect refers to a situation in which subtest *g*-loadings positively moderate a given effect size, indicating that *g* variance is the source of that effect size. Jensen effects manifest as positive correlations between the column vector of the effect sizes associated with each subtest (e.g. the strength of a subtest’s correlation with religiosity) and the column vector of their associated *g* loadings (Jensen 1998). The present study asks specifically whether the negative religion–IQ nexus is a Jensen effect. Finding that it is not (indeed it is an anti-Jensen effect), it explores possible explanations for the existence of this nexus, leading us to examine the role of autism spectrum traits.

## Method

To address the research question, we reanalysed two large datasets which looked at the relationship between religiousness and IQ in such a way that differences on *g* could be discerned: Verhage (1964), who employed a student sample, and a dataset presented in Steppan (2010). Both studies were analysed in Steppan (2010), who compared IQ scores, and tested for Jensen effects, in Protestant versus Catholic regions, in order to examine the Protestant Ethic (the high socioeconomic achievement of Protestants relative to Catholics). In contrast to Steppan, we went back to Verhage (1964) and included non-church members in our analysis. We first perform a Jensen effect analysis, using data from a number of previous studies. In general, *g* loadings were computed by conducting a principal-axis factor analysis on the correlation matrix of a test battery’s subtest scores. The subtests’ loadings on the first unrotated factor indicate the subtest’s loading on *g*. These *g* loadings were always matched to the age range of the groups involved in the comparison as closely as possible. If the age range of the comparison groups comprised more than one age group of the IQ battery, then weighted average *g* loadings were computed for all age groups of the IQ battery that fell within the age range of the comparison groups. Difference scores (*d*) were computed by taking the higher-scoring religious group’s mean IQ score and subtracting the mean IQ score of the lower-scoring religious group, and dividing by the pooled *SD*. Finally, Pearson correlations between the column vectors of the *d* scores and *g* loadings were computed. This correlation yields a moderation statistic, indicating the degree to which subtest *g* saturation moderates the associated effect size (*d*) in question (if at all).

### Corrections for Artefacts

Psychometric meta-analytical techniques (Hunter and Schmidt 1990) were applied using the software package developed by Schmidt and Le (2004). Those techniques are based on the principle that there are statistical artefacts in every dataset and that most of these artefacts can be corrected. In this case, we carried out a bare-bones meta-analysis, where we corrected for only one artefact, namely sampling error, which in the present study is reflected in the number of subtests in the IQ battery used. An IQ battery with 4 subtests has more sampling error than an IQ battery with 25 subtests.

### Choice of *SD*

For computing standardized effect sizes (*d*), from the mean differences between groups, the best estimate of *SD* available was used. Our choice of *SDs*, is, in order of preference (1): the *SD* of a national standardization sample; (2) the *SD* of a control group; and (3) a weighted average of the *SD* of the groups involved in the comparison. As neither the first or second option was available, the weighted average *SD* was used, computed as the pooled *SD: pooled SD *= √(((*N*_1_ − 1)*SD_1_^2^ + (*N*_2_ − 1)*SD_2_^2^)/(*N*_1_ + *N*_2_ − 2)) for two samples. For three samples, the pooled *SD* formula changes to *pooled SD* = √(((*N*_1_ − 1)*SD_1_^2^ + (*N*_2_ − 1)*SD_2_^2^ + (*N*_3_ − 1)*SD_3_^2^)/(*N*_1_ + *N*_2_ + *N*_3_ − 3)). For four or more samples, the formula changes in the same manner. When the samples have the same *N*, the pooled *SD* is equal to the ordinary average of the *SDs* (Agresti and Franklin 2007).

### Lack of Independence of Data Point in the Meta-Analytical Database

A potential problem for our study is that we use all datasets repeatedly for comparisons, which creates dependency between the data points. For instance, the Protestants are compared to the Catholics, but also to the non-religious. This is not optimal, because the software for meta-analysis assumes independence between the datasets.

An element of what is bothersome about using a dataset twice for a comparison is that peculiarities of that dataset will have an unwanted influence on two comparisons, so that the chance becomes smaller that this specific peculiarity is cancelled out by the other data points.

In most cases, an individual dataset in a meta-analysis has quite a bit of sampling error, because it is not a perfect sample from the population. However, in the case of the Dutch data, which make up most of the meta-analytical database, the GALO data were representative of the Dutch population and the subsamples were also quite representative for their specific religious group. So, an important element of lack of independence of datasets is that the samples used in a study show quite a bit of sampling error and may differ quite a bit from a representative sample. However, our religious samples are pretty representative of their respective populations, so there does not seem to be a significant problem with dependence of the Dutch samples.

Our approach to dependence of samples leans strongly on the approach by te Nijenhuis et al. (2016), who wrote:

“Schmidt and Hunter (2015, p. 437) state that when discussing dependence of samples, a distinction has to be made between arguments from statistical theory, and empirical outcomes of the strength of effects. According to statistical theory, if a small sample contributes a very large number of data points, then this may lead to undercorrection for sampling error in the meta-analysis. However, if the number of effect sizes contributed by each study is small in comparison to the total number of effect sizes then there is little error in the resulting accumulation …”

In the present study, each Dutch religious group is present in three out of seven comparisons, which is a clear indication of dependence in the database. However, none of the groups is small (Catholics: *N* = 666; Dutch Reformed Church: *N* = 460; Reformed Church Service: *N* = 148; and non-member of Church: *N* = 264), so there does not seem to be a problem. te Nijenhuis et al. (2016), also write:

“Several studies focused on the empirical question of how serious the problems created by violations of independence are in real data (see Bijmolt and Pieters 2001; Taveggia 1974; and Tracz et al. 1992). Schmidt and Hunter (2015, p. 452) conclude that the distortion caused by dependent samples is probably negligible.”

Taking all arguments into consideration, we decided to not correct for the at worst slight dependence in the samples.

## Results

The results of the studies on the correlation between *g* loadings and the score differences between different religious groups in the Netherlands—Catholics, Protestants, and non-religious—which have been shown to have different average IQs are shown in Table 1. The Protestant–Catholic division was motivated by previous studies clearly showing more nuanced levels of the religious/non-religious spectrum between these groups. Catholics, as a group, have consistently been found to be more religious, in terms of observance and belief, than Protestants in denominationally mixed nations as well as having lower average IQ or education levels as observed in England, Ireland, the USA, the Netherlands, and when comparing Protestant and Catholic areas in Europe (Pike 1869; Verhage 1964; Fichter 1952; Gorer 1955; Nyborg 2009; Meisenberg et al. 2012; Lynn and Vanhanen 2012). Table 1 reports data derived from one study, with participants numbering 10,497. It also lists the reference for the study, the specific groups being compared, cognitive ability test used, number of subtests in the IQ battery, vector correlation between *g* loadings and *d*, and the total sample size for the comparison of two religious groups. At first sight, the vector correlations between the subtests’ *g* loadings and the differences between the groups (in *d*) show no clear pattern with regard to magnitude and sign.

**Table 1:** Vector correlations between subtest *g* loadings and score differences (*d*) between different religious groups

Reference	Comparison	Test	*N*_subtests_	*r*	*N*_total_
Verhage (1964)	Roman Catholic—Dutch Reformed Church	GIT	10	− .57	544
	Roman Catholic—Reformed Church Service	GIT	10	.28	242
	Roman Catholic—Non-members of Church	GIT	10	− .49	378
	Dutch Reformed Church—Reformed Church Service	GIT	10	.80	224
	Dutch Reformed Church—Non-members of Church	GIT	10	− .09	335
	Reformed Church Service—Non-members of Church	GIT	10	− .61	190
Steppan (2010)	Roman Catholics—Protestants	EMS	9	− .22	8,959

*N*_total_ = total sample size; *r *= correlation *d* × *g.* The total sample size for the Verhage study is 1538

*GIT* Groninger intelligentie test [Groningen test of intelligence], *EMS* Eignungstest für das Medizinstudium [aptitude test for medical studies]

The mean IQs for the Dutch religious groups are: Roman Catholic 97.95, Dutch Reformed Church 99.85, Reformed Church Service 100.55, and Non-members of Church 103.80. The mean IQs for the Swiss religious groups are: Catholics 98.56 and Protestants 100.46

Table 2 presents the results of the bare-bones data synthesis of the seven data points. It shows the number of vector correlation coefficients (*K*), total sample size (*N*_total_), and the mean vector correlation and its standard deviation (*SD*_*r*_). The last column presents the percentage of variance explained by sampling errors (%VE). The analysis of all seven data points yielded an estimated aggregated vector correlation of − .13, with a relatively modest 47.1% of the variance in the observed correlations explained by sampling errors. It is clear that the comparison between the Dutch Reformed Church and Reformed Church Service is a statistical outlier with its very high value of *r* = .80. These two groups differ only .70 of an IQ point, which means their mean IQ scores are almost identical, supplying a good argument for leaving this data point out of the meta-analysis. The analysis of the remaining six data points yielded an estimated vector correlation of − .28 and now virtually all the variance between the data points is explained by sampling error (95.5). This means that the differences between religious groups are clearly not Jensen effects (this is in fact an anti-Jensen effect).

**Table 2:** Exploratory bare-bones data synthesis results for vector correlations between subtest *g* loadings and score differences (*d*) between different religious groups

Analysis	*K*	*N*_total_	Mean *r*	*SD*_*r*_	%VE
All	7	10,497	− .13	.33	47.1
Minus outlier	6	10,497	− .28	.31	95.5

Bare-bones data synthesis results: score differences between different religious groups (*d*), and subtest *g* loadings. *K* = number of data points; *N*_total_ = total sample size; mean *r* = mean of observed correlations corrected for sampling error; *SD*_r_ = standard deviation of observed correlations; %VE = percentage of variance accounted for by sampling errors. The total sample size does not change when the outlier is left out, as the two groups in the comparison are still used for other comparisons

The study on the relationship between IQ profiles of different religious groups and general intelligence was based on a very large sample size and yielded a small negative vector correlation, which clearly is not a Jensen effect. We therefore conclude that specialized cognitive abilities and test specificities may play a prospectively larger role in moderating the observed ability differences than *g*, when ethnically close-matched populations are used.

Given that the finding of an anti-Jensen effect seems to indicate that the differences between the groups are not on *g*, the key question is ‘What subtests may drive the intelligence differences?’ In Tables 3 and 4, the results of the subtests are provided. These results do not show a consistent pattern. In the Verhage (1964) study, the largest effect sizes are found on those tasks that involved naming animals and professions, and on word list, which seem to suggest that the subgroup differences depend particularly on vocabulary or verbal abilities. In the Steppan (2010) study, the largest group differences in the expected direction were on mental rotation and medical–scientific reasoning. The former, in particular, strongly relies on spatial abilities. Yet, there was a negative effect on mathematical ability.

**Table 3:** Subtests differences between the groups in Verhage (1964)

Sub test	Religious groups				Subtest differences between group (in effect size *d*)						Mean difference	*g* loading
	Roman Catholics (RC)	Dutch Reformed Church (DRC)	Reformed church services (RCS)	Non-church members (NoC)	RC-DRC	RC-RCS	RC-NoC	DRC-RCS	DRC-NoC	RCS-NoC		
					*d*	*D*	*d*	*d*	*d*	*d*	*M*_*d*_	
Word list	24.48 (*4.85*)	25.27 (*4.84*)	25.90 (*4.49*)	26.71 (*5.01*)	0.16	0.29	0.46	0.13	0.3	0.17	20	.75
Figures	24.76 (*5.03*)	24.88 (*4.61*)	25.54 (*5.34*)	25.78 (*4.98*)	0.02	0.16	0.21	0.13	0.18	0.05	.12	.78
Sailing exercise	24.58 (*5.14*)	25.12 (*4.80*)	25.84 (*4.89*)	25.33 (*4.24*)	0.11	0.25	0.15	0.14	0.04	− 0.1	.03	.79
Sorting	24.76 (*4.79*)	24.97 (*4.87*)	25.21 (*5.03*)	25.90 (*4.93*)	0.04	0.09	0.23	0.05	0.19	0.14	.13	.79
Gestalt completion	24.49 (*5.26*)	24.94 (*4.88*)	24.40 (*5.26*)	26.02 (*4.97*)	0.09	− 0.02	0.3	− 0.11	0.21	0.32	.14	.58
Numbers	24.31 (*4.99*)	25.54 (*4.95*)	25.27 (*5.21*)	25.93 (*4.67*)	0.25	0.19	0.33	− 0.05	0.08	0.13	.05	.48
Card rotations	24.95 (*4.97*)	24.82 (*5.08*)	24.73 (*5.38*)	25.66 (*5.20*)	− 0.03	− 0.04	0.14	− 0.02	0.17	0.18	.11	.75
Word matrix	24.46 (*5.17*)	25.06 (*5.05*)	25.72 (*4.49*)	25.60 (*5.49*)	0.12	0.25	0.22	0.13	0.11	− 0.02	.07	.80
Naming animals	24.55 (*5.02*)	24.91 (*4.18*)	25.15 (*4.88*)	26.38 (*5.23*)	0.07	0.12	0.36	0.05	0.29	0.24	.19	.64
Naming professions	24.40 (*5.23*)	25.03 (*4.92*)	24.73 (*4.75*)	26.26 (*5.43*)	0.12	0.06	0.36	− 0.06	0.24	0.3	.20	.60

To calculate the effect sizes, the pooled *SD* of the groups was used

**Table 4:** Subtest differences between Catholics and Protestants in Steppan (2010)

Subtest	Catholics	Protestants	*SD* (pooled)	*d*	*g* Loading
Find mistakes	98.56	100.46	10	.14	.74
Verbal memory	10.94	11.36	2.96	.15	.60
Figural memory	11.05	11.64	3.85	.15	.73
Mental rotation	12.20	12.71	3.36	.21	.60
Mathematical abilities	12.18	12.92	3.59	− .18	.73
Charts and tables	10.67	10.01	3.61	.15	.15
Medical–scientific reasoning	9.99	10.51	3.51	.19	.74
Text comprehension	10.15	10.78	3.35	.16	.72
Attention test	8.72	9.26	3.36	.10	.43

## Discussion

We tested the assumption inferred from the theories discussed in the introduction that the relationship between IQ differences between religious groups with different average IQs and their *g* loadings constitutes a Jensen effect, and may therefore indicate a role for *g* as a source of positive moderation of the group differences. As it clearly does not, when members of the same ethnic group are compared, evolutionary theories that purport to explain the weak, but robust negative religiousness–IQ nexus become somewhat less convincing. We would have expected, therefore, that a specific pattern of specialized cognitive abilities would have driven the negative religious–IQ nexus. However, contrary to our expectations, the analyses of the subtests differences did not reveal a clear pattern with regard to which specific cognitive abilities may drive the IQ differences between the different groups. In Verhage (1964), the largest differences seemed to occur on vocabulary. In the Steppan (2010) study, the subtest effect sizes showed less variation than in the Verhage study, but the largest effect sizes in the expected direction were found on mental rotation and medical–scientific reasoning. In the Steppan (2010) sample, the presumably more religious group did relatively better on mathematical reasoning.

A possible way of making sense of our findings is through the influence of autism spectrum disorder (ASD). There is a growing body of research on the negative relationship between ASD and religiosity. The evidence indicates, overall, that ASD is negatively associated with religious belief and that empathy is the mediating factor: autism, in part, may actually cause people to be less religious (see the systematic literature review by Dutton et al. 2018). Caldwell-Harris et al. (2011) studied discussions by 192 different contributors on an autism website, from which they were able to discern the views on religion held by the contributors. High-functioning autistic (HFA) individuals significantly demonstrated the highest rates of ‘non-belief identities’: Atheism (26%) and Agnosticism (17%). In the neurotypical (NT) group used as non-autistic controls, 17% were atheists and 10% were agnostics. The same authors conducted a survey with a sample of 61 people who self-identified as autistic. They found that those who regarded themselves as atheists scored significantly higher than those who were believers did on the Autism Quotient Scale, an instrument that quantifies the extent of autism. Barnes and Gibson (2013) found that those who had undergone religious experiences had elevated empathy, contrary to those with ASD. Jack et al. (2016) found that ‘moral concern’, which is also conceivably lower in those with ASD, predicted strength of religious belief and was negatively associated with analytic thinking. This implies that low religiousness is predicted by analytic thinking—which those with ASD are particularly adept at. Norenzayan et al. (2012) showed that autism predicted reduced religious belief, based on Canadian and American samples. Importantly, they found that it was the ability to mentalize that mediated the negative relationship between autism and religious belief. Lowicki and Zajenkowski (2016, 2017) and Vonk and Pitzen (2017) note that aspects of ASD—such as low emotional intelligence—are negatively associated with religious belief. Again, these are the aspects of ASD that relate to the ability to develop a sound theory of mind. The only counter-study of which we are aware is Reddish et al. (2016), which did not find any significant difference in religious behaviour or belief between an HFA group and an NT control group. However, this was based on a very small sample of 21 people.

So, all available studies with reasonable sample sizes are consistent with the notion that theory of mind is an important factor in the association between ASD and religious belief. Autistics tend to perceive the world in a mechanistic fashion, as a system. Accordingly, they should not perceive the complexity of the world as the workings of a sentient being, because they are unable to think about or even notice mental states. In this regard, they stand in stark contrast with schizotypal personality. Schizophrenia (a particularly pronounced manifestation of schizotypal personality) is associated with being extremely religious, as well as with belief in the paranormal and in conspiracy theories (see Dutton et al. 2018). This is because schizophrenics are so highly attuned to inferring mental states from external markers that they perceive evidence of mental states even in the world itself; it is as if the world has feelings and meaning; thus, schizophrenics routinely experience the presence of God. There are a variety of models which have attempted to make sense of religious experiences or the feeling that there is a god. Azari et al. (2005) used brain scans to conclude that religious experience is primarily a cognitive phenomenon rather than an emotional one. Religious experience, they concluded, relates to neural processes involved in ‘relational cognitivity’—thinking about relationships. Schjoedt et al. (2009) assessed which areas of the brain were active when participants engaged in informal prayer compared to when wishing to Santa Claus. They found that brain activity during prayer more closely resembled that which occurred while talking to a real person than to an imaginary figure. Religious experience appears to involve the ability to empathize with somebody else. Although it is currently small, the extant brain imaging evidence indicates that religious experience involves brain areas that are associated with mentalizing and relating to other people.

This raises the question of how people with ASD perform in IQ tests. There is no clear direction to these results. A literature review by Ghaziuddin and Mountain-Kimchi (2004) reported that some studies have found that those with Asperger’s syndrome—a middle-level ASD—have high-level verbal IQ and but can be deficient on performance IQ. Other studies have revealed that those with pronounced ASD, such as high-functioning autism, show a reversal in this pattern: poor verbal IQ and high mathematical IQ. Consistent with this, Karpinski et al. (2018) have recently presented evidence that highly intelligent people seem to manifest autism traits, in terms of an enhanced tendency to systematize and a diminished ability to empathize (see also Baron-Cohen 2002 and Crespi 2016). Tests of the IQ of HFA persons have found that their scores are high in fluid intelligence, in other words on matrix and similarities reasoning subtests (e.g. Hayashi et al. 2008). Dawson et al. (2007) have shown that HFA persons score strongly on Raven’s (a matrix test) relative to broader IQ test batteries, on average 30 percentile points, and in some cases 70 percentile points higher than they score on the WISC. However, it is not high-functioning autism (HFA) which predicts low religiosity but rather ASD more broadly, and here the IQ profile pattern is much less clear cut. In this regard, a recent study found that possessing genetic risk factors for ASD was associated with logical memory, verbal intelligence and *g*, meaning it would confer a small advantage in IQ tests even in the absence of greater *g* (Clarke et al. 2016). So, such an explanation would potentially make sense of the otherwise difficult to explain results which we find. However, more research must be conducted to discover why we did not find any positive moderating effect of *g*. Our finding that the more religious scored better on Mathematics would seem to be consistent with the results of some studies which have found deficiencies on performance IQ among those with ASD.

It should be cautioned that it is likely that the findings of our analysis only hold for within-population comparisons, where the differences in *g* might be expected to be relatively small. If we were to make comparisons between populations, such as between Middle Eastern Muslims and European Catholics, it is probable that, in line with Spearman’s hypothesis, the differences would indeed be on *g.* Moreover, insofar as the groups that are being compared in the present study may not be precisely equal in terms of *g,* there is room for small group differences in *g* to attenuate the anti-Jensen effect which we have found. But, naturally, it would be very difficult to find two groups that were precisely equal in terms of *g.* A second limitation is that our results are not comprehensive. However, there are two substantial datasets and, for this reason, our results are likely to generalize to religious differences in other countries. A third limitation is that the Steppan dataset makes comparisons between regions rather than individuals or groups, which leaves room for anomalies. Finally, it is worth cautioning that our ASD explanation for apparent oddities in our results is paralleled by similar anomalies in terms of the IQ profile of those with normal range IQ who have an ASD ranging all the way up to HFA. This may lead us to question the conceptual validity of ASD, or how accurately we can measure it, something which others have already done (e.g. Lundqvist and Lindner 2017; Ghaziuddin and Mountain-Kimchi 2004).
